# Gender Differences in Amplitude of Low-Frequency Fluctuation Alterations in Healthy Volunteers by Acupuncture on Left “LI 15”: A Resting-State fMRI Study

**DOI:** 10.1155/2024/3986094

**Published:** 2024-10-28

**Authors:** Guoyan Li, Yinghua Jing, Jing Ren, Song Cui, Ding Yu

**Affiliations:** ^1^Department of Rehabilitation Medicine, Guangzhou First People's Hospital, School of Medicine, South China University of Technology, Guangzhou, China; ^2^Department of Neurology, RWTH Aachen University, Aachen, Germany; ^3^Department of Radiology, Guangzhou First People's Hospital, School of Medicine, South China University of Technology, Guangzhou, China

**Keywords:** acupuncture, functional magnetic resonance imaging, gender difference, *Jianyu*, low-frequency fluctuation, poststroke shoulder pain

## Abstract

**Objectives:** This study is aimed at evaluating gender differences in neural activity change response to the acupuncture on left *Jianyu* (LI 15) in healthy volunteers.

**Methods:** Forty healthy volunteers (20 males and 20 females) received 20-min acupuncture on left LI 15 and underwent functional magnetic resonance imaging (fMRI) scans before and after acupuncture. Amplitude of low-frequency fluctuations (ALFF) in the 0.01–0.08 Hz range were determined for both scans. Paired *t*-tests were performed on ALFF between two scans separately for the male and female groups to identify neural changes related to acupuncture.

**Results:** After acupuncture, males showed significantly increased ALFF in the left cerebellum and right angular gyrus but decreased ALFF in the left precentral gyrus, left inferior occipital gyrus, and right fusiform gyrus. However, the ALFF change in females is almost negligible.

**Conclusions:** Brain functional activity in response to acupuncture on left LI 15 is noticeably different between males and females. This is preliminary evidence that gender may be an important factor for optimal clinically personalized acupuncture therapy for poststroke shoulder pain in the future.

## 1. Introduction

Poststroke shoulder pain is a common complication that severely affects the rehabilitation process of stroke patients [1]. As a traditional Chinese medicine therapy, acupuncture is effective for the treatment of chronic musculoskeletal, headache, and osteoarthritic pain with therapeutic effects persisting over time [2–6]. In terms of acupuncture therapy in poststroke shoulder pain, *Jianyu* (LI 15), belonging to the large intestine meridian, is widely and frequently used in combination with other acupoints in clinical treatment [7, 8]. Additionally, a Chinese meta-analysis showed that LI 15 is the preferred acupuncture point for the treatment of poststroke shoulder pain [9]. Nevertheless, the neural mechanisms underlying acupuncture analgesia on LI 15 are still unclear.

Functional magnetic resonance imaging (fMRI) provides a noninvasive method to investigate the neural activity associated with acupuncture [10–12]. The amplitude of low-frequency fluctuations (ALFF) is a reliable and replicable method for measuring low-frequency blood oxygen level–dependent (BOLD) signals, which reflect regional spontaneous neural activity in resting-state fMRI [13]. Compared to other fMRI analysis methods, the ALFF has the advantage of being data-driven and not influenced by any prior hypothesis. Besides, ALFF also provides a broader exploration of the whole brain at the voxel level. ALFF alterations were commonly reported in previous acupuncture studies. For example, acupuncture for migraine patients normalized the reduced ALFF in the rostral ventromedial medulla/trigeminocervical complex [14]; ankle acupuncture for chronic low back pain was significantly associated with decreased ALFF in the cerebellum and insula [15]; and electroacupuncture for acute pain after total knee arthroplasty resulted in enhanced ALFF in the precuneus [16]. However, to our knowledge, few neuroimaging studies have focused on gender effects in acupuncture for analgesic treatment, although gender differences exist in clinical treatment outcomes of acupuncture therapy.

Thus, we selected left LI 15 as the single acupoint and aimed to explore specific brain regions corresponding to this acupoint and assess gender differences in neural activity changes to this acupoint in healthy volunteers. Our findings would give insights into gender-specific acupuncture treatment for patients with poststroke shoulder pain and contribute specific stimulation targets to improve the efficacy of the combination of acupuncture and other brain modulation techniques (such as transcranial direct current stimulation).

## 2. Materials and Methods

### 2.1. Participants

Forty healthy volunteers (20 males/20 females) were recruited for this study. All participants were right-handed; with regular diets and normal sleeping patterns; and had no history of chronic diseases, neurological or psychiatric disorders, alcohol abuse, or shoulder trauma. Additionally, participants were excluded if they had a skin broken around the left shoulder bone, or if they had acupuncture therapy in the 3 months before the study.

This study was approved by the Ethics Committee of Guangzhou's First People's Hospital (Approval No. S-2023-154-03). Each volunteer was fully introduced to the experiment and all signed informed consent forms.

### 2.2. Experiment Design

**Figure 1:**
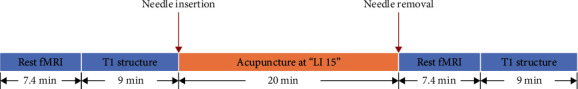
Procedure diagram for acupuncture and imaging scans. The fMRI scans were performed twice before and after acupuncture, and each scan included resting-state functional and structural images.

As shown in Figure 1, this study consisted of two main parts: acupuncture and image acquisition. fMRI scans (each including resting-state functional and structural image scans) were focused on the BOLD signals before acupuncture and after 20 min of acupuncture, respectively. All experiments were carried out at Guangzhou's First People's Hospital, Guangdong, China.

### 2.3. Acupuncture Intervention

**Figure 2:**
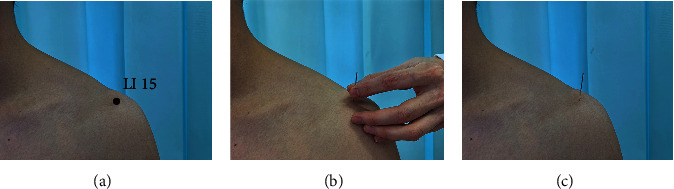
Acupuncture schematic. (a) Location of LI 15; (b) Needle insertion time point. (c) Needle retention in shoulder acupuncture.

The acupoint LI 15 (shown in Figure 2(a)), located on the deltoideus of the shoulder, is in the depression of the anterior superior portion of the shoulder. Acupuncture was performed by an experienced acupuncturist (J.R.) on LI 15 of the left shoulder. The disposable silver acupuncture needles (0.22 × 25 mm; Dongbang Brand, Suzhou, China) were applied in the present study. After sterilizing the skin at the acupoint LI 15, the acupuncturist held the needle and rapidly inserted it into the subcutaneous tissue at an angle of about 90° to the surface of the body (Figure 2(b)) and then placed the needle down to the skin and pushed it toward the shoulder bone until the needle was inserted into the subcutis of about 13–20 mm (alternatively, the acupoint was reached when the participant gave feedback on a sense of slightly localized soreness and numbness), leaving it in place for 20 min (Figure 2(c)). Participants would not experience any discomfort during the period of needle retention.

### 2.4. MRI Data Acquisition

All MRI data were acquired using a 3.0-T MRI scanner (Siemens, Erlangen, Germany) with an eight-channel head coil by an expert technician (S.C.). During the data acquisition, each participant was informed to lie quietly, stay relaxed and awake with closed eyes, and remain as motionless as possible. Foam paddings and earplugs were also provided to minimize head movements and to reduce noise, respectively.

The parameters of a gradient reflection echo (GRE) sequence for resting-state fMRI data were as follows: 33 axial slices, slice thickness/gap = 4.0/0 mm, repetition time (TR) = 2000 ms, echo time (TE) = 21 ms, flip angle (FA) = 78°, voxel size = 3.5 × 3.5 × 4.0 mm, field of view (FOV) = 224 × 224 mm, 220 volumes, and 7.4 min.

The parameters of a 3D T1-weighted sequence for structural images were as follows: 176 transversal slices, slice thickness/gap = 1.0/0 mm, TR = 2530 ms, TE = 2.93 ms, FA = 9°, voxel size = 1.0 × 1.0 × 1.0 mm, FOV = 256 × 256 mm, and 9 min.

### 2.5. Data Preprocessing

Resting-state fMRI data were processed using SPM12 (http://www.fil.ion.ucl.ac.uk/spm) and RESTplus V1.24 [17] in MATLAB (Version R2019a, MathWorks, Inc., Natick, Massachusetts, United States). The preprocessing steps include the following: (1) removing the first 10 time points in NIFTI data format to ensure signal stability; (2) slice timing using the middle slice as the reference to correct acquisition delay; (3) head movement correction using the first slice as a reference; (4) spatial normalization to the Montreal Neurological Institute (MNI) space using tissue segmentation from T1-weighted structure image (resampling voxel size = 3.0 × 3.0 × 3.0 mm); (5) spatial smoothing with a 6-mm full width at half maximum (FWHM) Gaussian kernel; (8) removing the linear trend; (9) regressing out the head motion effect (using Friston 24 parameters) [18], as well as white matter and cerebrospinal fluid signal.

Head motions of all participants were less than 2.0 mm of translation or 2° of rotation in any direction of *x*, *y*, or *z*. The quality of all images was good enough to allow for the next analysis.

### 2.6. ALFF Calculation

ALFF is an indicator of spontaneous neuronal activity in the resting state [13]. After preprocessing, the unfiltered data were transformed into frequency domain power spectra based on fast Fourier transforms. The square root of each frequency was calculated in the power spectrum, and the average square root across 0.01–0.08 Hz at each voxel was defined as the ALFF value. For further data standardization, the ALFF of each voxel was divided by the global mean of ALFF as the mALFF value.

### 2.7. Statistical Analysis

Paired *t*-tests were applied to compare the mALFF between pre- and postacupuncture via RESTplus V1.24 [17]. The resultant T-maps were corrected for multiple comparisons based on Gaussian random field (GRF), and the significant level was set at voxel *p* < 0.001 and cluster *p* < 0.05 (two-tailed test). Additionally, results with *p* < 0.001 and cluster size > 10 voxels (uncorrected) were provided if GRF-corrected results were not available.

## 3. Results

### 3.1. Demographic Characteristics

Forty volunteers all completed the experiment, and demographic information is presented in Table 1. There was no significant difference between male and female groups in age and education. No significant difference in needling sensation was reported between the two groups.

### 3.2. Male Group

**Figure 3:**
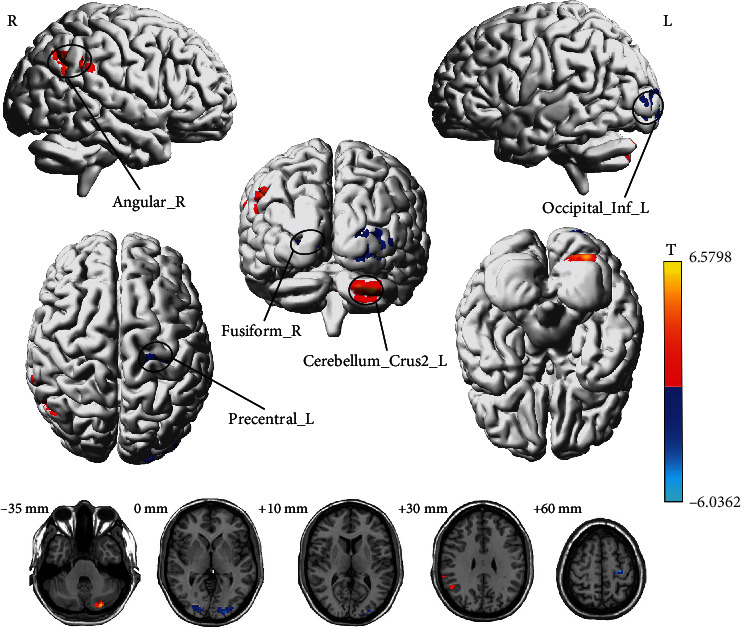
T-maps of brain functional changes for the male group pre- and postacupuncture on left LI 15. Red/blue colors indicate increased/decreased ALFF values after acupuncture, respectively (voxel *p* < 0.001, cluster *p* < 0.05, GRF correction, two-tailed test). Details are described in Table 2.

Compared to the baseline, male participants showed significantly increased ALFF in Left Cerebellum Crus 2 and right angular gyrus, whereas decreased ALFF values were observed in the left precentral gyrus, left inferior occipital gyrus, and right fusiform gyrus after acupuncture (GRF correction, voxel *p* < 0.001, cluster *p* < 0.05, two-tailed test) (Figure 3 and Table 2).

### 3.3. Female Group

**Figure 4:**
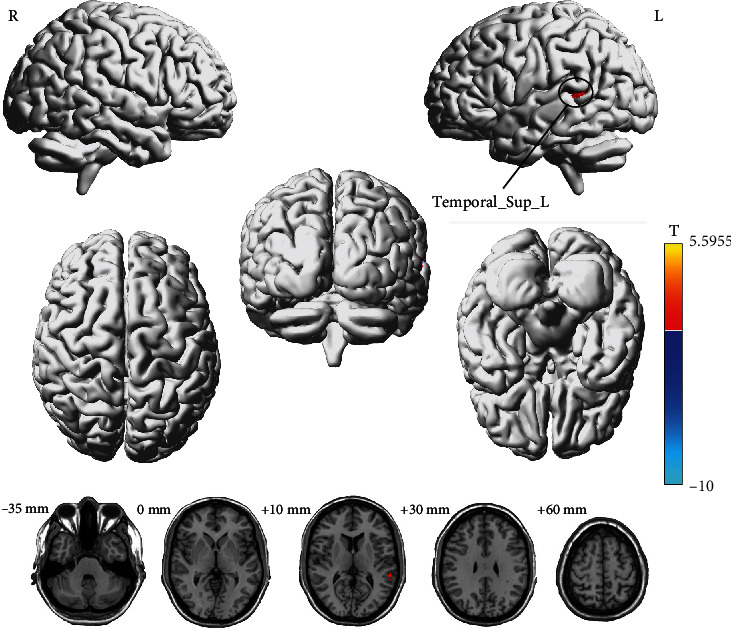
T-maps of brain functional changes for the female group pre- and postacupuncture on left LI 15. Red/blue colors indicate increased/decreased ALFF values after acupuncture, respectively (uncorrected, *p* < 0.001, cluster size > 10 voxels). Details are described in Table 3.

There was no significant result in the female group after GRF correction. But females presented an increased ALFF in the left superior temporal gyrus after acupuncture (uncorrected, *p* < 0.001, cluster size > 10 voxels) (Figure 4 and Table 3).

## 4. Discussion

The present study has shown remarkable neural differences between healthy males and females after acupuncture intervention on left LI 15. Compared with the period before acupuncture stimulation, males exhibited decreased ALFF responses in the left precentral, inferior occipital, and right fusiform gyrus but increased ALFF in Left Cerebellum Crus 2 and the right angular gyrus. However, there was only a small change in the left temporal lobe in females. These results may contribute to understanding the acupuncture mechanism and facilitate precise acupuncture treatment strategies based on gender.

### 4.1. Male Group

After 20 min of acupuncture, the low-frequency oscillatory signals in the left precentral motor cortex (BA6) and bilateral occipital cortex (BA18) were reduced in male volunteers. Multiple neuroimaging studies have consistently shown that pain can lead to brain hyperactivation, including the precentral and occipital gyrus [19, 20]. On the one hand, the motor cortex adopts a layer-specific pathway to modulate sensory and aversive-emotional components of neuropathic pain, which achieves pain alleviation [21–23]. On the other hand, the occipital cortex can integrate information from the somatosensory system, vision, and hearing and is important in the interoceptive processing of pain relief [24]. Our findings support a potential mechanism of acupuncture to suppress hyperactivation in the motor–occipital regions to reduce pain. However, further studies on patients with poststroke shoulder pain are needed to confirm it.

In addition, acupuncture caused an increase in neural activity in the cerebellum and the angular gyrus (BA39). The cerebellum is typically considered to be a brain region engaged in motor processing, but a growing body of studies indicates that it also plays a critical role in perception and regulation beyond the motor domain [25, 26]. In line with the results of the two previous studies on brain activation after acupuncture, male volunteers had enhanced activity in the left cerebellum although the acupoints and the acupuncture duration were inconsistent [27, 28]. Furthermore, animal experiments have shown that acupuncture treatment could activate endogenous pain control systems by stimulating relevant acupoints, thereby modulating Transient Receptor Potential Vanillic 1 and related molecular pathways in the cerebellum for pain relief [29, 30]. The current finding further establishes that the cerebellum is important in acupuncture treatment for analgesia. Regarding the angular gyrus, the mechanism of its increased activity in acupuncture analgesia is more complex. The angular gyrus is located at the junction of the parietal, temporal, and occipital lobes. It serves as a common hub in various functional networks, such as pain management, cognitive function, and emotional regulation [31]. On the one hand, the angular gyrus plays a crucial role in pain pathways. Its increased activation may be related to the neuroplastic changes in the brain, which were promoted by acupuncture [32]. On the other hand, the increased ALFF in the angular gyrus might also be a secondary effect of experimental stimulation. For example, acupuncture typically provokes a characteristic sensation of numbness and soreness when the needles are inserted into the body [33]. Although this sensation is too subtle and short-lived to be ignored by the subjects, the effects induced in the brain would not disappear quickly. This sensation may prompt the angular gyrus to receive and integrate inputs from the somatosensory cortex in conscious response [34, 35]. Additionally, it is worth noting that the angular gyrus, especially in the right hemisphere, helps in directing attention by integrating visual, auditory, and tactile inputs. This enables the brain to combine various sensory modalities, improving focus and understanding in complex environments. Damage to the right angular gyrus can result in attention deficits [36]. During the fMRI scanning, volunteers might be in a state of hyperconcentration, thereby engaging neural activity in the right angular gyrus.

Altogether, the current findings provide new insights into the neural mechanism of acupuncture on left LI 15 modulating the premotor–cerebellar loop. This circuit is worthy of further validation in follow-up patients with poststroke shoulder pain.

### 4.2. Female Group

In contrast to the male group, the females did not show significant changes in brain activity after acupuncture. Since one meta-analysis study by Jia et al. pointed out that strictly multiple comparison correction with small *p* values may fail to yield robust results [37], we also tested the female group by applying the uncorrected threshold (individual voxel *p* < 0.001, cluster size > 10 voxels, edge connected, and uncorrected). However, even without correction, only the left superior temporal gyrus (BA22) showed slightly increased activity after acupuncture. A previous study implicated that connectivity between the thalamus and temporal cortex plays a significant role as a neural mechanism in acupuncture analgesia [24]. In the present study, while there was an indication of temporal-related changes in the female group, the small change was only shown in an uncorrected threshold and did not survive under the strict multiple correction, which might be due to the small sample, limiting the statistical power to generalize this finding. Though the female group's findings do not give us a hint of the specific brain activity involved in acupuncture and the possible neural pathways, it is certain that the same acupuncture affects completely different brain activity in males and females. Studies on chronic pain indicated the gender gap in shoulder/neck pain, that women scored higher in severity, extent, and duration of pain than men [38], and that women seek acupuncture treatment more frequently than men [39]. These all suggest that differences exist in systems-level mechanisms of pain processing and acupuncture for analgesia between males and females. One possibility is that sex hormones differ between males and females [40–42]. Sex hormones can directly or indirectly alter the distribution of neurotransmitter receptors and regulate neuropeptide expression and cholinergic activity through many cellular and molecular processes. These influence the function of neural response systems, resulting in gender differences in sensitive processing such as mood, cognitive function, and pain [43]. Therefore, our findings suggest that it is important for acupuncture therapy to consider gender in its treatment strategy. Particularly in female patients, a longer duration of acupuncture or multiacupoints should be considered to improve the efficacy of the treatment.

### 4.3. Strengths and Limitations

The strength of this study is that this is the first to explore the gender effects of acupuncture for shoulder pain treatment. Besides, peripheral and central sensitization of acupoints is present in patients with shoulder pain [8]. Thus, specific acupoints are important in acupuncture. The present study focused on single-acupoint acupuncture to precisely investigate the neural mechanisms of acupuncture on left LI 15.

There are some limitations of this study. Firstly, the sample size is small with only 20 subjects in each group. To reduce false-positive results, we performed a multiple comparison correction on the results, but this resulted in weak functional changes in the female group being harder to discover. Secondly, this study was only conducted on healthy volunteers, hence it is necessary to take this fully into account when understanding the findings. Future studies with large samples of patients with poststroke shoulder pain are warranted to validate the results and determine the optimal gender-specific treatment of acupuncture. Finally, it is valuable to assess bilateral single-point acupuncture at the same time to better unravel the neural mechanisms of acupuncture on LI 15.

## 5. Conclusions

The present study demonstrates that acupuncture on left LI 15 has a distinctly different modulation pattern of neural activity in healthy males and females and it is important to consider gender effects in its acupuncture treatment. To meet the requirements of precision medicine strategies, further research in patients with poststroke shoulder pain is necessary to determine the optimal gender-specific acupuncture based on different neural mechanisms in males and females.

## Figures and Tables

**Table 1:** Demographic information of healthy volunteers.

**Characteristics**	**Male group**	**Female group**
Sample of size	20	20
Age: mean ± SD (years)	21.7 ± 0.66	21.25 ± 0.85
Educational level: mean ± SD (years)	14.7 ± 0.66	14.25 ± 0.85

Abbreviation: SD = standard deviation.

**Table 2:** Brain regions of statistically different ALFF values for the male group after acupuncture on left LI 15.

**Peak location (AAL)**	**BA**	**Cluster size**	**Peak ** **T** ** value**	**Peak MNI coordinate (mm)**		
				**X**	**Y**	**Z**
Cerebellum_Crus2_L	NA	50	6.5798	−27	−84	−36
Angular_R	39	41	5.6941	51	−54	33
Precentral_L	6	26	−5.66	−30	−21	60
Occipital_Inf_L	18	116	−6.0362	−24	−90	−6
Fusiform_R	18	46	−5.4262	27	−87	−3

*Note:* Cluster size = the number of voxels.

Abbreviations: AAL = Automated Anatomical Labeling, BA = Brodmann's area, MNI = Montreal Neurological Institute, NA = not available.

**Table 3:** Brain regions of statistically different ALFF values for the female group after acupuncture on left LI 15.

**Peak location (AAL)**	**BA**	**Cluster size**	**Peak ** **T** ** value**	**Peak MNI coordinate (mm)**		
				**X**	**Y**	**Z**
Temporal_Sup_L	22	12	5.5955	−57	−45	12

*Note:* Cluster size = the number of voxels.

Abbreviations: AAL = Automated Anatomical Labeling, BA = Brodmann's area, MNI = Montreal Neurological Institute, NA = not available.

## Data Availability

The relevant data in this study are available from the corresponding author upon reasonable request.
